# Higher uric acid serum levels are associated with sarcopenia in west China: a cross-sectional study

**DOI:** 10.1186/s12877-022-02817-x

**Published:** 2022-02-12

**Authors:** Xiaolei Liu, Xiaoyan Chen, Fengjuan Hu, Xin Xia, Lisha Hou, Gongchang Zhang, Xuchao Peng, Xuelian Sun, Shuyue Luo, Jirong Yue, Birong Dong

**Affiliations:** 1 National Clinical Research Center for Geriatrics, West China Hospital, Sichuan University, No. 37, Guo Xue Xiang Renmin Nan Lu, Chengdu, 610041 Sichuan Province China; 2 Geriatric Health Care and Medical Research Center, Sichuan University, Chengdu, Sichuan Province China; 3 Zigong Mental Health Center, Sichuan Province, China; 4 Department of Geriatrics, West China Hospital, Sichuan University, No. 37, Guo Xue Xiang, Chengdu, 610041 Sichuan China

**Keywords:** Uric acid (UA), Handgrip strength (HGS), Skeletal muscle index (SMI), Sarcopenia

## Abstract

**Background:**

Sarcopenia is the decline in muscle strength and mass attributed to aging. The pathogenesis of sarcopenia may be triggered by oxidative stress and uric acid (UA) has strong antioxidant properties. The aim of this study was to investigate the relationship between UA and sarcopenia in community-dwelling adults of West China using the baseline data of West China Health and Aging Trend (WCHAT) study.

**Design:**

A cross-sectional study.

**Methods:**

4236 adults aged 50 years or older in communities of west China were enrolled in this study. We applied Asian Working Group for Sarcopenia (AWGS) 2019 criteria to define sarcopenia. Muscle mass was measured using skeletal muscle index (SMI) based on bioimpedance analysis (BIA). Handgrip strength (HGS) and gait speed (GS) were recorded, respectively. Different variables like anthropometry measures, life styles, chronic disease and blood test were collected. General linear model was done to investigate the relationship between UA and HGS/GS/SMI, adjusting age, ethnic groups, sleeping quality, education level, cognitive function, smoking history, drinking history, ADL score, and chronic disease.

**Results:**

Participants were grouped according to UA quartiles by gender. After adjusting for potential confounders, a negative association between serum UA levels and sarcopenia was shown both in men and women. And a significant association between serum UA levels and HGS in women was shown as an inverted J shape. Besides, a positive association between the UA quartiles and SMI was observed, irrespective of gender.

**Conclusions:**

Our results showed that higher uric acid levels were significantly correlated with higher muscle mass and grip strength among Chinese adults aged over 50. Higher UA serum levels might slow down the progression of sarcopenia.

## Introduction

Sarcopenia was an age-dependent loss of muscle mass and function which was common among older adults, leading to disability, loss of independence and death [1]. The prevalence of sarcopenia varies in different countries according to different diagnostic criteria. In west China, our previous studies showed a high prevalence of sarcopenia which was 19.31% in 4500 participants over 50 years old [2]. According to recent studies, sarcopenia was significantly associated with ethnicity, age, gender, obesity, life styles, chronic disease and so on [3]. In addition to these risk factors, age-related decreases in hormone concentrations could cause loss of muscle mass and strength, such as growth hormone, testosterone, thyroid hormone, vitamin D, albumin and insulin-like growth factor [4]. Another metabolic factor, uric acid (UA), was studied most recently in the relationship with skeletal muscle mass and/or strength and another metabolic factor, uric acid (UA), was studied most recently in the relationship with skeletal muscle mass and/or strength, but the conclusions were varied and ambiguous[5, 6, 8, 9].

As the final product of purine metabolism, UA is generated in the xanthine/hypoxanthine reactions and other potentially deleterious prooxidant molecules are produced as a by-product of this reaction. As a result of this, UA has been treated as a reliable marker of oxidative stress [7]. UA is a crucial endogenous antioxidant, which can eliminate reactive oxygen species (ROS) and, thus preventing oxidative stress. Recently, it was found that UA was positively associated with muscle mass and strength in kidney transplant patients [6]. Besides, another cross-sectional study showed that higher serum UA levels may be associated with better hand grip strength among Chinese adults aged over 45 [5]. However, in a study of 586 Japanese men aged over 30, it was found that hyperuricemia was associated with reduced muscle strength, and UA levels showed an inverted J-shaped curve with handgrip strength [8]. What’s more, in a sample of 7,544 US men and women aged 40 and above, it showed that for every unit (mg/dL) increase in uric acid, the odds ratio of manifesting a skeletal muscle mass index at least one standard deviation below the reference mean was 1.12. Participants in the highest grouping (> 8 mg/dL) of serum uric acid concentration had 2.0 times the odds of manifesting sarcopenia compared to the lowest grouping (< 6 mg/dL) after adjusting for the additional covariates [9].

Unfortunately, there are few studies on the association of serum UA levels and sarcopenia in west China. We speculated that serum UA was negatively associated with sarcopenia and higher UA was associated with better grip strength or muscle mass, particularly in old adults. To explore this hypothesis, we grouped the participants according to UA quartiles by gender. Then we performed our study to determine the relationship between sarcopenia and UA in a large group of multi-ethnic residents enrolled in the West-China Health and Aging Trend Study (WCHAT). Specifically, we also investigated the relationship between UA and skeletal muscle index (SMI), grip strength (HGS) and gait speed (GS).

## Materials and methods

### Study sample

**Fig. 1:**
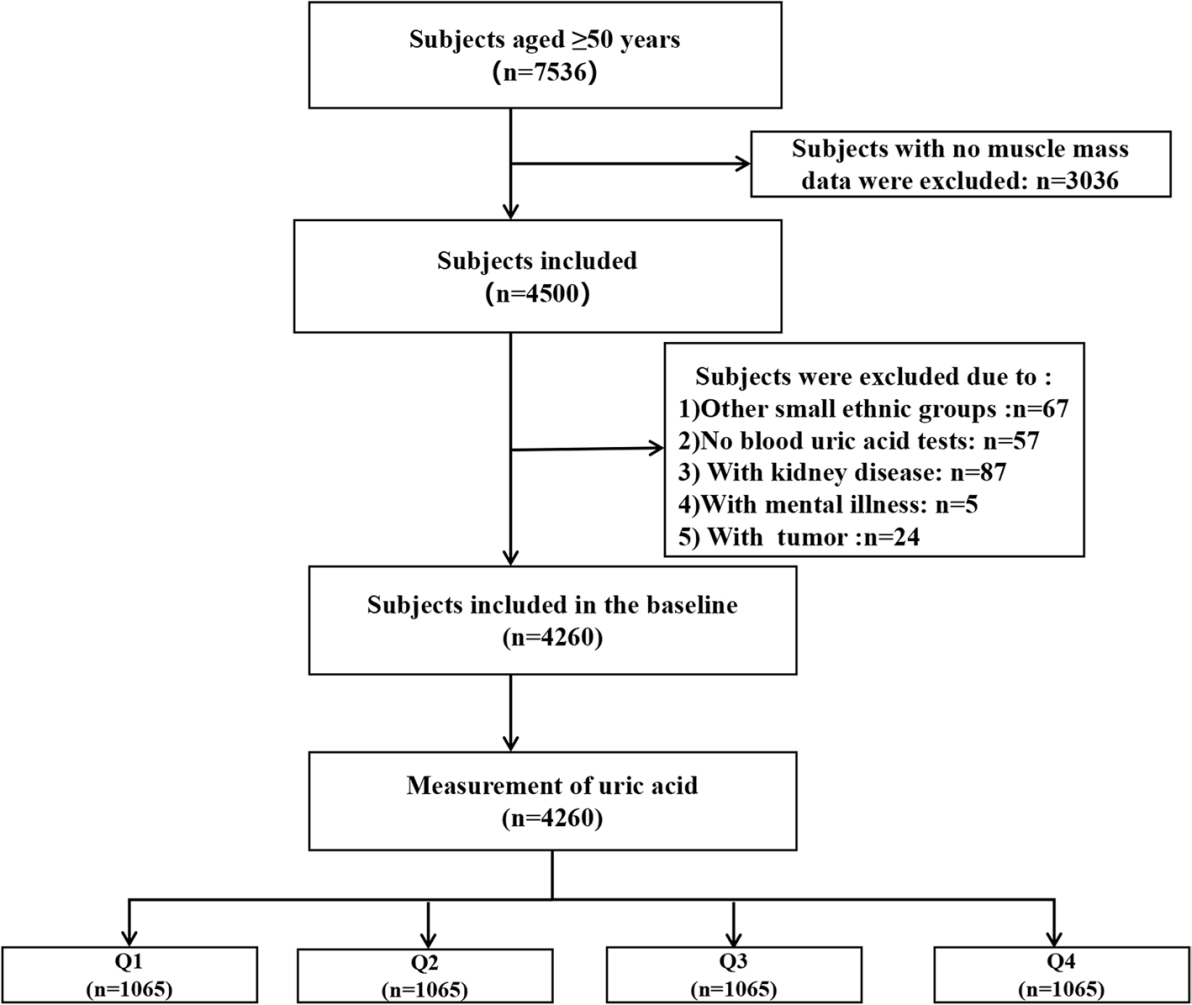
Flow chart of study participants. Initially, we recruited 7536 community-dwelling multi-ethnic residents in total. 4500 participants did the bioelectrical impedance analysis (BIA) which is available for the selection of sarcopenia. Then other small ethnic group participants (*n* = 67), participants without blood uric acid test (*n* = 57), participants with kidney disease (*n* = 87), participants with mental disease (*n* = 5) and participants with tumor (*n* = 24) were excluded. Finally, 4260 participants were included and were grouped according to UA quartiles in our study

The current research is a cross-sectional analysis obtaining baseline data of the WCHAT study between July 2018 and October 2018 [10]. Participants aged ≥ 50 years were selected from 4 provinces including Yunnan, Guizhou, Sichuan, and Xinjiang. Participants were recruited by convenience and asked verbally by the researchers about their willingness to take part in the study. Before investigation, informed consent was signed and obtained by each participant. Initially, we recruited 7536 community-dwelling multi-ethnic residents in total. 4500 participants did the bioelectrical impedance analysis (BIA) which is available for the selection of sarcopenia. Then other small ethnic group participants (*n* = 67), participants without blood uric acid test (*n* = 57), participants with kidney disease (*n* = 87), participants with mental disease (*n* = 5) and participants with tumor (*n* = 24) were excluded. Finally, 4260 participants were included and were grouped according to UA quartiles in our study (Fig. 1).

### Definitions of sarcopenia

We defined sarcopenia according to the AWGS 2019 [11, 12] which defined sarcopenia according to low skeletal muscle mass, low strength, and/or low physical function. Skeletal muscle mass was estimated by a trained doctor using a bioimpedance analysis (BIA) device (InBody 770, Biospace, Korea). For AWGS 2019, the SMI was using BIA predicted skeletal muscle mass and cutoffs were 7.0 kg/m^2^ in men and 5.7 kg/m^2^ in women. Grip strength was measured using a dynamometer (EH101; Camry, Zhongshan, China) to test the muscle strength. Tests were performed on two independent occasions using the dominant hand and the largest value was recorded. Cutoffs of grip strength was defined as 28 kg in men and 18 kg in women. The physical function was estimated using gait speed (GS) through a 4-m walking test. The walking time was recorded using a kind of infrared sensor and the acceleration phase was strictly excluded. The participants were asked to perform the test by walking at a normal pace. Subjects stood at the starting point and upon the starting command, walked forward at a normal pace to the 4-m line. During the test, subjects wore common shoes, could use mobility aids, but could not be assisted. There were no time limits to the assessments and subjects could stop and rest if necessary. Sitting down was prohibited. The participants performed 2 trials, and the results were averaged to the nearest 0.01 m/s. The cutoff of gait speed was defined as less than 1.0 m/s.

### Demographic data and blood sample collection

Information regarding age, gender, ethnic groups, education level, smoking history and alcohol consumption history was gathered. Blood samples were drawn from the vein in the morning after a minimum of 8 h of fasting. Blood handling and collection was carried out under strictly standardized conditions. UA level were measured using the same standard.

### Assessment of cognition, depression, sleep quality and chronic diseases

Cognitive status was assessed using a 10-item Short Portable Mental Status Questionnaire (SPMSQ) [13] and the result was based on educational level. Depressive symptoms were assessed using the 15‐item Geriatric Depression Scale (GDS-15) [14]. Sleeping quality was assessed using the Pittsburgh Sleep Index Scale (PSQI) questionnaire [15]. A medical history of chronic disease was self-reported. These disease conditions included hypertension, diabetes mellitus, coronary heart disease (CHD), liver disease, chronic obstructive pulmonary disease (COPD), gastrointestinal disease, stroke and osteoarticular disease.

### Statistical analyses

The data analyses were performed using SPSS version 20.0 (SPSS Inc, Chicago, IL). The categorical data was presented as counts (percentages), and the normal distributed continuous data was presented as mean (standard deviation [SD]). We grouped the participants to different quartile boundaries in men and women, separately (In female, Q1^#^, < 253.85umol/l, 253.85 ≤ Q2^#^ < 293.8umol/l, 293.8 ≤ Q3^#^ < 340.4umol/l, 340.4umol/l ≤ Q4^#^; In male, Q1* < 319.2umol/l, 319.2 ≤ Q2* < 372.25umol/l, 372.25 ≤ Q3* < 423.525umol/l, 423.525umol/l ≤ Q4*). For continuous variables, one-way ANOVA was used to detect differences across groups for the continuous variables, and Fisher’s Least Significant Difference (LSD) post hoc analysis was used to determine the difference between every two groups. For the categorical variables, the chi-squared test was used to detect the difference across groups. When significant difference was identified across groups, column proportions tests (z-tests) with Bonferroni correction were performed to determine the difference between every two groups. During most testing, *p* < 0.05 was considered statistically significant, however, p-values were corrected for z-tests with the Bonferroni correction (with the statistical significance set at *p* < 0.008, where 0.008 = 0.05/6). We also performed UA subgroup analyses according to gender. The relationship between UA and sarcopenia was estimated by deriving odds ratios (ORs) and 95% confidence intervals (CIs) from multivariate logistic regression models. General linear model was done to investigate the relationship between UA and HGS/GS/SMI, adjusting age, ethnic groups, GDS score, sleeping quality, education level, cognitive function, smoking history, drinking history, ADL score, and chronic disease(hypertension, diabetes, coronary heart disease, chronic obstructive pulmonary disease, osteoarthropathy, liver disease, gastrointestinal disease, stroke history). A value of P < 0.05 (two-side) was considered to be statistically significant.

## Results

### Characteristics of participants

We included 4260 participants (1542 men and 2718 women). Participants was grouped according to quartiles of UA in male and female, separately. Table 1 shows the association of UA quartiles with participants' characteristics in the whole sample. The higher of the UA level, the higher mean age of the participants in female, while the lower mean age in male. Besides, the higher of the UA level, the higher percentage of drinking and smoking in both male and female. The prevalence of hypertension in the Q4 group was significantly higher than the Q1 group in both male and female. While the percentage of diabetes in the Q4 group was significantly higher than the Q2 and Q3 group in female. And the percentage of sarcopenia was significantly lower in the Q4 group than the Q1 group in both male and female. Specifically, with the increasing level of UA, the SMI increase in both male and female.

**Table 1:** Baseline characteristics of participants according to the quartiles of UA

Characteristics	Uric Acid (umol/L) Male(*n* = 1542)					Uric Acid (umol/L) Female(*n* = 2718)				
	Q1*	Q2*	Q3*	Q4*	P	Q1#	Q2#	Q3#	Q4#	P
Age (years),mean (SD)	64.28(7.54)^d^	63.89(8.03)	63.63(8.42)	62.65(8.53)^a^	0.037	61.51(7.86)^d^	60.88(7.77)^d^	60.89(7.81)^d^	63.15(8.95)^abc^	< 0.01
Ethnic groups (%)					0.084					< 0.01
Han	161(41.92)	136(35.14)	148(38.34)	161(41.82)		275(40.5)^d^	287(42.27)	326(48.01)	345(50.66)^a^	
Zang	110(28.65)	140(36.18)	130(33.68)	135(35.06)		189(27.84)	173(25.48)	162(23.86)	165(24.23)	
Qiang	99(25.78)	87(22.48)	92(23.83)	79(18.18)		176(25.92)	194(28.57)	160(23.56)	131(19.24)	
Yi	14(3.65)	24(6.2)	16(4.15)	19(4.94)		39(5.74)	25(3.68)	31(4.57)	40(5.87)	
Education (%)					0.57					< 0.01
No formal education	62(16.9)	67(18.77)	70(18.92)	61(16.62)		285(44.74)^c^	225(34.99)	210(32.16)^a^	266(40.86)	
Primary school	154(41.96)	135(37.82)	146(39.46)	132(35.97)		182(28.57)	217(33.75)	218(33.38)	182(27.96)	
Middle school and above	151(41.14)	155(43.42)	154(41.62)	174(47.41)		170(26.69)	201(31.26)	225(34.46)	203(31.18)	
smoking history(%)					0.784					0.244
No	210(57.38)	192(53.93)	208(56.68)	202(55.04)		622(97.8)	623(97.8)	636(97.7)	623(96.29)	
Yes	156(42.62)	164(46.07)	159(43.32)	165(44.96)		14(2.2)	14(2.2)	15(2.3)	24(3.71)	
drinking history(%)					0.186					0.46
No	204(55.74)	202(56.74)	191(52.04)	182(49.59)		563(88.52)	545(85.56)	563(86.48)	563(87.02)	
Yes	162(44.26)	154(43.26)	176(47.96)	185(50.41)		73(11.48)	92(14.44)	88(13.52)	84(12.98)	
ADL score, mean (SD)	99.01(3.81)	98.74(5.42)	99.22(2.69)	99.35(2.66)	0.149	99.06(4)	99.38(2.47)	99.15(3.23)	98.82(3.68)	0.028
GDS score, mean (SD)	2.4(2.13)	2.61(2.5)	2.45(2.23)	2.17(1.92)	0.065	2.98(2.49)	2.8(2.41)	2.66(2.42)	2.7(2.41)	0.091
Cognitive function (%)					0.026					0.013
No decline	342(93.7)	313(87.92)	340(93)	336(91.55)		511(80.47)	537(84.17)	536(82.59)	545(84.5)	
Mild decline	18(4.93)	36(10.11)	16(4.37)	26(7.08)		86(13.54)	76(11.91)	97(14.95)	68(10.54)	
Moderate-severe decline	5(1.37)	7(1.97)	10(2.73)	5(1.37)		38(5.99)	25(3.92)	16(2.47)	32(4.96)	
Sleep quality (%)					0.793					0.547
Good	196(54.14)	191(52.47)	193(52.45)	202(55.65)		345(53.82)	327(50.39)	345(531.16)	328(51.01)	
Bad	166(45.86)	173(47.53)	175(47.55)	161(44.35)		196(46.18)	322(49.61)	304(46.84)	315(48.99)	
Hypertension (%)					< 0.01					< 0.01
No	314(81.77)^d^	308(79.59)	281(72.8)	263(68.31)^a^		567(83.51)^d^	556(81.89)	521(76.73)	464(68.14)^a^	
Yes	70(18.23)^d^	79(20.41)	105(27.2)	122(31.69)^a^		112(16.49)^d^	123(18.11)	158(23.27)	217(31.86)^a^	
Diabetes (%)					0.25					< 0.01
No	346(90.1)	364(94.06)	355(91.97)	354(91.95)		639(94.11)	649(95.58)^d^	649(95.58)^d^	613(90.01)^bc^	
Yes	38(9.9)	23(5.94)	31(8.03)	31(8.05)		40(5.89)	30(4.42)^d^	30(4.42)^d^	68(9.99)^bc^	
CHD					0.041					0.04
No	373(97.14)	378(97.67)	377(97.67)	364(94.55)		661(2.65)	670(98.67)	661(97.35)	655(96.18)	
Yes	11(2.86)	9(2.33)	9(2.33)	21(5.45)		18(2.65)	9(1.33)	18(2.65)	26(3.82)	
Liver disease (%)					0.091					0.307
No	366(95.31)	379(97.93)	376(97.41)	367(95.32)		672(1.03)	665(97.94)	669(98.53)	666(97.8)	
Yes	18(4.69)	8(2.07)	10(2.59)	18(4.68)		7(1.03)	14(2.06)	10(1.47)	15(2.2)	
Gastrointestinal disease (%)					0.476					0.615
No	361(94.01)	373(96.38)	365(94.56)	365(94.81)		631(92.93)	629(92.64)	636(93.67)	642(94.27)	
Yes	23(5.99)	14(3.62)	21(5.44)	20(5.19)		48(7.07)	50(7.36)	43(6.33)	39(5.73)	
Stoke history (%)					0.808					0.565
No	376(97.92)	382(98.71)	378(97.93)	377(97.92)		670(98.67)	669(98.53)	673(99.12)	669(98.24)	
Yes	8(2.08)	5(1.29)	8(2.07)	8(2.08)		9(1.33)	10(1.47)	6(0.88)	12(1.76)	
COPD (%)					0.735					0.176
No	380(98.96)	380(98.19)	380(98.45)	381(98.96)		676(99.56)	675(99.41)	670(98.67)	672(98.68)	
Yes	4(1.04)	7(1.81)	6(1.55)	4(1.04)		3(0.44)	4(0.59)	9(1.33)	9(1.32)	
Osteoarticular disease(%)					0.83					0.294
No	358(93.23)	363(93.8)	361(93.52)	355(92.21)		614(90.43)	619(91.16)	631(92.93)	615(90.31)	
Yes	26(6.77)	24(6.2)	25(6.48)	30(7.79)		65(9.57)	60(8.84)	48(7.07)	66(9.69)	
Sarcopenia(%)					< 0.01					< 0.01
No	254(66.15)^d^	271(70.03)^d^	292(75.65)	314(81.56)^ab^		541(72.52)^d^	645(78.37)	798(78.31)	1319(78.89)^a^	
Yes	130(33.85)^d^	116(29.97)^d^	94(24.35)	71(18.44)^ab^		205(27.48)^d^	178(21.63)	221(21.69)	353(21.11)^a^	
SMI,kg/m^2^,mean (SD)	7.18(0.76)^cd^	7.3(0.81)^d^	7.41(0.79)^ad^	7.57(0.79)^abc^	< 0.01	6.04(0.73)^bcd^	6.24(0.68)^a^	6.26(0.75)^a^	6.3(0.79)^a^	< 0.01
Handgrip strength,kg,mean (SD)	27.83(8.66)	28.16(9.22)	28.42(9.65)	29.25(0.82)	0.212	17.63(5.24)^bc^	18.43(5.58)^a^	18.73(5.63)^a^	`18.34(5.76)	0.003
Gait speed,m/s,,mean (SD)	0.87(0.29)	0.88(0.28)	0.87(0.27)	0.87(0.24)	0.933	0.84(0.28)	0.85(0.26)	0.86(0.27)^d^	0.81(0.27)^c^	0.009

*Note*: Baseline characteristics of participants according to the quartiles of UA. For continuous variables, one-way ANOVA was used to detect differences across groups for the continuous variables, and Fisher’s Least Significant Difference (LSD) post hoc analysis was used to determine the difference between every two groups. For the categorical variables, the chi-squared test was used to detect the difference across groups. When significant difference was identified across groups, column proportions tests (z-tests) with Bonferroni correction were performed to determine the difference between every two groups. During most testing, *p* < 0.05 was considered statistically significant, however, p-values were corrected for z-tests with the Bonferroni correction (with the statistical significance set at *p* < 0.008, where 0.008 = 0.05/6). Q stands for UA: Q1 is the lowest quartile and Q4 is the highest quartile. ^a^Significantly different from the Q1 group. ^b^Significantly different from the Q2 group. ^c^Significantly different from the Q3 group. ^d^Significantly different from the Q4 group. CHD, coronary heart disease; COPD, chronic obstructive pulmonary disease. In male: Q1* < 319.2umol/l, 319.2umol/l ≤ Q2* < 372.25umol/l, 372.25umol/l ≤ Q3* < 423.525umol/l, 423.525umol/l ≤ Q4*.In female: Q1# < 253.85umol/l, 253.85umol/l ≤ Q2# < 293.8umol/l, 293.8umol/l ≤ Q3# < 340.4umol/l, 340.4umol/l ≤ Q4#

### Serum UA level and sarcopenia

Table 2 showed the relationship between UA quartiles and sarcopenia in non-adjusted model and adjusted model in female and male, respectively. We grouped UA levels according to gender. In non-adjusted model, sarcopenia was negatively associated with Q3* (OR 0.629, 95%CI 0.459–0.861) and Q4* level in male (OR 0.442, 95%CI 0.317–0.616), and negatively associated with Q2^#^(OR 0.666, 95%CI 0.514–0.864),Q3^#^ (OR 0.653, 95%CI 0.504–0.848) and Q4^#^(OR 0.645, 95%CI 0.497–0.837) in female. Q1 was regarded as the baseline category. In adjusted model which adjusted age, ethnic groups, GDS score, sleeping quality, education level, cognitive function, smoking history, drinking history, ADL score, and chronic disease(hypertension, diabetes, coronary heart disease, chronic obstructive pulmonary disease, osteoarthropathy, liver disease, gastrointestinal disease, stroke history), sarcopenia was still significantly associated with Q3* (OR 0.664, 95% CI 0.462–0.955) and Q4* (OR 0.513, 95%CI 0.349–0.753) group in male. Besides, in adjusted model, sarcopenia was significantly associated with UA in female with a dosage effect (Q2^#^, OR 0.729, 95% CI 0.542–0.982; Q3^#^, OR 0.593, 95% CI 0.436–0.805; Q4^#^, OR 0.477, 95% CI 0.348–0.652).

**Table 2:** The relationship between UA quartiles and sarcopenia in non-adjusted model and adjusted model

variable		Non-adjusted Model		Adjusted Model	
		*P*-value	OR (95% *CI*)	*P*-value	OR (95% *CI*)
Male	Q1*	-	-	-	-
	Q2*	0.248	0.836(0.618–1.133)	0.572	0.903(0.633–1.288)
	Q3*	0.004	0.629(0.459–0.861)	0.027	0.664(0.462–0.955)
	Q4*	< 0.001	0.442(0.317–0.616)	0.001	0.513(0.349–0.753)
Female	Q1^#^	-	-	-	-
	Q2^#^	0.002	0.666(0.514–0.864)	0.037	0.729(0.542–0.982)
	Q3^#^	0.001	0.653(0.504–0.848)	0.001	0.593(0.436–0.805)
	Q4^#^	0.001	0.645(0.497–0.837)	< 0.001	0.477(0.348–0.652)

*Note*:Adjusted model:adjusted age, ethnic groups, GDS score, sleeping quality, education level, cognitive function, smoking history, drinking history, ADL score, and chronic disease(hypertension, diabetes, coronary heart disease, chronic obstructive pulmonary disease, osteoarthropathy, liver disease, gastrointestinal disease, stroke history). In female: Q1^#^ < 253.85umol/l, 253.85umol/l ≤ Q2^#^ < 293.8umol/l, 293.8umol/l ≤ Q3^#^ < 340.4umol/l, 340.4umol/l ≤ Q4^#^; In male: Q1* < 319.2umol/l, 319.2umol/l ≤ Q2* < 372.25umol/l, 372.25umol/l ≤ Q3* < 423.525umol/l, 423.525umol/l ≤ Q4*. The odds ratios (OR) represent the odds of sarcopenia with the first quartile of UA as the baseline category

### Serum UA level and SMI/GS/HGS

The results of general linear models for the relationship between UA levels and SMI/GS/HGS in male and female were presented in Table 3. Q1 was regarded as the baseline category. We found that serum UA level was independently positively associated with SMI in female in Q2^#^(β 0.201, 95% CI 0.119–0.283), Q3^#^ (β 0.248, 95% CI 0.167–0.330) and Q4^#^ group(β 0.270, 95% CI 0.189–0.352) with a dosage effect. But UA level was negatively associated with GS in female in the Q4 group (β-0.039). Besides, the serum UA levels showed an inverted J-shaped relationship with HGS in female (Q2^#^, β 0.737, Q3^#^, β 1.142, Q4^#^, β 0.694). While in male, the UA levels was positively associated with SMI in Q3*(β 0.237, 95%CI 0.12–0.354) and Q4* group(β 0.359, 95%CI 0.241–0.476). And UA levels was positively associated with HGS in the Q4* group in male (β 1.406, 95% CI 0.018–2.794).

**Table 3:** General linear model testing the relationship between UA quartiles and SMI, gait speed, handgrip strength after adjusting for relevant confounders

variable	β	SE	*P*-value	95%CI	
**In female**					
**SMI (kg/m**^**2**^**)**					
Q1^#^(Ref)	-	-	-	-	-
Q2^#^	0.201	0.0418	< 0.001	0.119	0.283
Q3^#^	0.248	0.0415	< 0.001	0.167	0.33
Q4^#^	0.27	0.0417	< 0.001	0.189	0.352
**Gait speed (GS) (m/s)**					
Q1^#^(Ref)	-	-	-	-	-
Q2^#^	0.004	0.0156	0.817	-0.027	0.034
Q3^#^	0.01	0.0155	0.512	-0.02	0.041
Q4^#^	-0.039	0.0156	0.012	-0.07	-0.008
**Handgrip strength (HGS) (kg)**					
Q1^#^(Ref)	-	-	-	-	-
Q2^#^	0.737	0.3212	0.022	0.107	1.366
Q3^#^	1.142	0.3194	< 0.001	0.516	1.768
Q4^#^	0.694	0.3207	0.03	0.066	1.323
**In male**					
**SMI (kg/m**^**2**^**)**					
Q1*(Ref)	-	-	-	-	-
Q2*	0.088	0.0599	0.144	-0.03	0.207
Q3*	0.237	0.0598	< 0.001	0.12	0.354
Q4*	0.359	0.0605	< 0.001	0.241	0.476
**Gait speed (GS) (m/s)**					
Q1*(Ref)	-	-	-	-	-
Q2*	0.01	0.0205	0.642	-0.031	0.05
Q3*	0.005	0.0203	0.821	-0.035	0.044
Q4*	0.006	0.0203	0.757	-0.034	0.046
**Handgrip strength (HGS) (kg)**					
Q1*(Ref)	-	-	-	-	-
Q2*	0.233	0.7151	0.745	-1.169	1.635
Q3*	0.482	0.7068	0.495	-0.903	1.867
Q4*	1.406	0.7083	0.047	0.018	2.794

*Note*: General linear model was adjusted age, ethnic groups, GDS score, sleeping quality, education level, cognitive function, smoking history, drinking history, ADL score, and chronic disease(hypertension, diabetes, coronary heart disease, chronic obstructive pulmonary disease, osteoarthropathy, liver disease, gastrointestinal diseas, stroke history). In female: Q1^#^ < 253.85umol/l, 253.85umol/l ≤ Q2^#^ < 293.8umol/l, 293.8umol/l ≤ Q3^#^ < 340.4umol/l, 340.4umol/l ≤ Q4^#^; In male: Q1* < 319.2umol/l, 319.2umol/l ≤ Q2* < 372.25umol/l, 372.25umol/l ≤ Q3* < 423.525umol/l, 423.525umol/l ≤ Q4*

## Discussion

We found that UA was negatively associated with sarcopenia after adjusted confounding factors. Besides, serum UA levels shared a significant inverted J-shaped curve relationship with HGS in female and only a positive relationship in male in Q4 group. And we found that UA level was also significantly associated with SMI, irrespective of gender. Collectively, these results suggest that UA seems to be a factor in protecting the muscle mass and the strength of the lower limbs. However, we cannot ignore the fact that this study was only a cross-sectional study. The relationship between uric acid and sarcopenia does not imply causality.

Our study was consistent with previous studies [5–9]. One study in China showed that higher uric acid levels were significantly correlated with higher muscle mass, grip strength in 388 participants aged over 60 [16]. Another cross-sectional study in China which included 3,079 middle-aged and older participants indicated a positive association between UA and ASMI which was tested by dual-energy X-ray absorptiometry [17]. Moreover, a most recent prospective cohort study reported that a higher baseline UA levels still remained significantly associated with higher follow-up strength measures during a 3-year follow-up period [18]. These studies supported that maintaining optimal levels of serum UA may help to maintain the quality and strength of skeletal muscle.

In our study, we found a gender difference of the relationship between UA and HGS and this could be related with hormone difference. Several studies reported that estrogen promotes UA secretion and leads to increases in UA levels in postmenopausal women [19], which may partially strengthen the significant association between UA and ASM in the total female population. However, another study also found that UA was positively associated with ASM and this association was only significant in males but not in females [20]. And in men with T2DM, higher serum UA was found to be an independent risk factor of reduced muscle mass [21]. Future studies should be performed to evaluate whether the association between UA and muscle mass/strength is sex-dependent.

The exact mechanism that explains the positive associations of UA with muscle mass and strength remains unclear. This might be related with the powerful antioxidant capacity of UA which may protect skeletal muscle function from ROS-induced protein oxidative damage [22]. Besides, uric acid was affected by protein and carbohydrate intake, and lower uric acid levels were associated with poor nutrition and weight loss. And UA was an important risk factor associated with poor grip strength [23]. Moreover, elevated serum UA levels were positively correlated with serum creatinine level which was correlated to the individual’s muscle mass [24]. Higher UA levels was found to be positively associated with the rate of normalized protein equivalent of nitrogen appearance [25], which was shown to be beneficial for skeletal muscle mass. Extracellular UA has antioxidant effects, acting as a powerful scavenger of free radicals, protecting muscle cell from oxidative damage [26]. UA was shown to have potential therapeutic effects in suppressing the redox process, promoting both myoblast proliferation and differentiation in muscle aging [27]. And some research found that higher plasma uric acid protects neurons from toxic effects of peroxynitrite in Parkinson’s disease and multiple sclerosis [28, 29]. All of these studies indicated that serum UA may have a protective role in aging-associated decline in muscle strength [30].

On the contrary, higher uric acid predicts obesity, diabetes and chronic kidney disease (CKD) which were all associated with the development of sarcopenia [31]. So maybe a very high level of uric acid was not beneficial for sarcopenia. Severe sarcopenia might be associated with low uric acid, since poor nutrition often correlates with aging sarcopenia and low uric acid levels [32]. And when people develop progressive diseases, such as Alzheimer’s disease, nutrition gets worse and uric acid falls [33]. Since our study included most relatively “healthy” participants, the level of UA was not very much high. Another research found that UA was not a major factor controlling oxidative stress in vivo and plasma urate provides little protection from oxidants [34]. And it was well known that an increased UA level was related to high inflammatory cytokines such as IL-6, CRP and TNF-α, which were contributors to poor muscle strength [35]. Besides, a study found that sarcopenia might be treated using allopurinol, a medicine which could reduce the serum UA level [36]. Whether uric acid play a protective role in sarcopenia need a longitudinal study, maybe after a period of 10 years or more.

Some limitations of our study need to be considered. Firstly, people over 50 years old were enrolled in this study and most of them were in the age range of 50–60 years old. The relationship between UA level and sarcopenia could only reflect mostly middle-aged participants. Secondly, although we included many important confounders in our study. More adjustment should be made for confounders. For example, gout and medicine intake which might affect the UA level should be adjusted. Besides, we used 4-m gait speed evaluation instead of 6-m gait speed and this might exist some bias. Lastly, our study was only an observational cross-sectional study which cannot determine the causal relationship between sarcopenia and serum UA level. Further longitudinal research or clinical trial research was needed.

## Conclusions

In conclusion, our findings showed that higher UA serum levels was related with better HGS/SMI, not with GS. And sarcopenia was negatively associated with UA with a dosage effect. Besides, there existed some gender difference when these relationships were studied in men and women separately.

## Data Availability

The datasets generated and analyzed during the current study will be available two years later and is also available now from the corresponding author on a reasonable request.
